# The eEF1γ Subunit Contacts RNA Polymerase II and Binds Vimentin Promoter Region

**DOI:** 10.1371/journal.pone.0014481

**Published:** 2010-12-31

**Authors:** Nicoletta Corbi, Enrico Maria Batassa, Cinzia Pisani, Annalisa Onori, Maria Grazia Di Certo, Georgios Strimpakos, Maurizio Fanciulli, Elisabetta Mattei, Claudio Passananti

**Affiliations:** 1 Istituto di Biologia e Patologia Molecolari CNR, c/o Regina Elena Cancer Institute, Rome, Italy; 2 Department of Experimental Medicine, University of L'Aquila, L'Aquila, Italy; 3 Istituto di Neurobiologia e Medicina Molecolare, Consiglio Nazionale delle Ricerche (CNR), IRCCS Fondazione S. Lucia, Rome, Italy; 4 Department of Therapeutic Programs Development, Regina Elena Cancer Institute, Rome, Italy; Victor Chang Cardiac Research Institute, Australia

## Abstract

Here, we show that the eukaryotic translation elongation factor 1 gamma (eEF1γ) physically interacts with the RNA polymerase II (pol II) core subunit 3 (RPB3), both in isolation and in the context of the holo-enzyme. Importantly, eEF1γ has been recently shown to bind Vimentin mRNA. By chromatin immunoprecipitation experiments, we demonstrate, for the first time, that eEF1γ is also physically present on the genomic locus corresponding to the promoter region of human Vimentin gene. The eEF1γ depletion causes the Vimentin protein to be incorrectly compartmentalised and to severely compromise cellular shape and mitochondria localisation. We demonstrate that eEF1γ partially colocalises with the mitochondrial marker Tom20 and that eEF1γ depletion increases mitochondrial superoxide generation as well as the total levels of carbonylated proteins. Finally, we hypothesise that eEF1γ, in addition to its role in translation elongation complex, is involved in regulating Vimentin gene by contacting both pol II and the Vimentin promoter region and then shuttling/nursing the Vimentin mRNA from its gene locus to its appropriate cellular compartment for translation.

## Introduction

The RNA polymerase II (pol II) core enzyme consists of at least twelve different subunits that associate with several mediator proteins and general transcription factors to form the holoenzyme complex [1]-[4]. We had previously cloned two subunits of the human pol II enzyme, RPB11 (UniProtKB: P52435) and RPB3 (UniProtKB: P19387) [5]–[6]. RPB3 and RPB11 form a heterodimer that is reminiscent of the α subunit homodimer of bacterial RNA polymerase that is involved in promoter recognition. The RPB3/RPB11 heterodimer plays a central role in the interaction between pol II and the mediator complex, suggesting functional conservation from prokaryotes to eukaryotes [7]. Using the RPB3 subunit as bait in a series of yeast two-hybrid experiments, we defined RPB3 involvement in tissue-specific transcription. We demonstrated that RPB3 directly contacts several transcription factors, including ATF4, a member of the ATF/CREB family and Myogenin, a member of the MyoD gene family [8]–[9]. In addition, we have recently shown that RPB3 is retained/stored in the cytoplasm interacting with CCHCR1, the psoriasis vulgaris candidate gene product [10].

Here, we show, for the first time, that RPB3, alone and complexed in pol II, interacts with the Eukaryotic Elongation Factor 1 subunit gamma (eEF1γ) (UniProtKB: P26641) that is a part of eEF1 complex. Eukaryotic elongation factor 1 (eEF1) is a macromolecular complex that catalyses the transfer of aminoacyl-tRNAs to ribosomes [11]. In higher eukaryotes, eEF1 consists of three or four subunits, eEF1α, eEF1β, eEF1γ and eEF1δ, respectively renamed eEF1A, eEF1Bα, eEF1Bγ and eEF1Bδ [11]–[12]. For the purposes of simplicity in this article we use the older nomenclature (eEF1γ). The eEF1α subunit of EF1 binds aminoacyl-tRNA in a GTP-dependent manner and the resulting ternary complex binds to the ribosome [13]. Following aminoacyl-tRNA binding to the ribosomal A site via a codon-anticodon interaction, GTP is hydrolysed to GDP. Subsequently, GDP remains bound to eEF1α and eEF1β acts as nucleotide exchange factor, regenerating eEF1α-GTP for the successive elongation cycle. The physiological role of the eEF1γ subunit in this context is still not well defined. There is some evidence that eEF1γ stimulates, but is not required for, the nucleotide exchange activity of eEF1β [12]. Indeed, eEF1γ appears dispensable for translation, its absence doesn't seem to affect global rate of translational elongation. Instead eEF1γ depletion in *Saccharomyces cerevisiae* provides resistance to oxidative stress [14]. A role of eEF1γ in the oxidative stress response pathways is justified by the presence in the N terminus of eEF1γ of a conserved sequence resembling the glutathione-binding region of the theta class of Glutathione S-transferases (GST) enzymes, which is involved in the detoxification of oxygen radicals [15]. The over-expression of eEF1γ, described in several tumours, influences tumour aggressiveness presumably by altering the redox balance [12], [16]. Nevertheless, multiple/additional roles for eEF1γ are emerging, some of which can be regulated by phosphorylation driven by several protein kinases [17]–[18]. eEF1γ displays an affinity for membrane and cytoskeleton elements and it could properly anchor the different subunits of the EF1 complex to the cytoskeleton [12], [19]–[21]. Interestingly, Al-Maghrebi et al. (2002) showed in studies in vitro and in vivo that eEF1γ binds the 3′UTR of Vimentin (UniProtKB: P08670) mRNA, demonstrating for the first time the RNA-binding properties of eEF1γ [22]. In addition, human eEF1γ was recently identified in a proteomic screen as a member of the pre-mRNA 3′ end cleavage complex [23]. To further reinforce a role for eEF1γ in RNA metabolism, Fan et al. demonstrated in Drosophila that eEF1γ activity is required for the viability of both the whole animal and individual cells, emphasising that recessive lethality occurs during early larval development. This observation is consistent with an extensive maternal contribution of the RNA and protein to the embryo [18]. In this scenario eEF1γ could contribute to the anchoring and translation of a set of mRNAs that are preferentially translated on cytoskeletal- or membrane-bound ribosomes, such as Vimentin mRNA.

Vimentin is the major intermediate filament protein involved in cell structural support, signal transduction and organelle positioning [24]. Morris et al. (2000) demonstrated that misdirecting Vimentin mRNA it is possible to alter cell morphology and motility [25]. The replacement of the Vimentin 3′ untranslated region (3′UTR), where both eEF1γ and Hax1 proteins bind, with the βactin 3′UTR resulted in the misdirection of Vimentin mRNA. In particular, Vimentin has been recently reported to have a regulatory role in supporting the morphology, organization and function of mitochondria [26]. Here, we show that eEF1γ interacts with pol II and stays on/binds Vimentin gene promoter, where eEF1γ may start to perform its function/s. In addition, we show that eEF1γ partially localises within mitochondria and that eEF1γ depletion induces the incorrect compartmentalisation of Vimentin protein, resulting in a severe compromise of cellular shape and mitochondria localisation. We show that eEF1γ depletion increases mitochondrial generation of superoxide and total carbonylated proteins. Finally, we hypothesise that eEF1γ plays an additional role in RNA metabolism.

## Results

**Figure 1:**
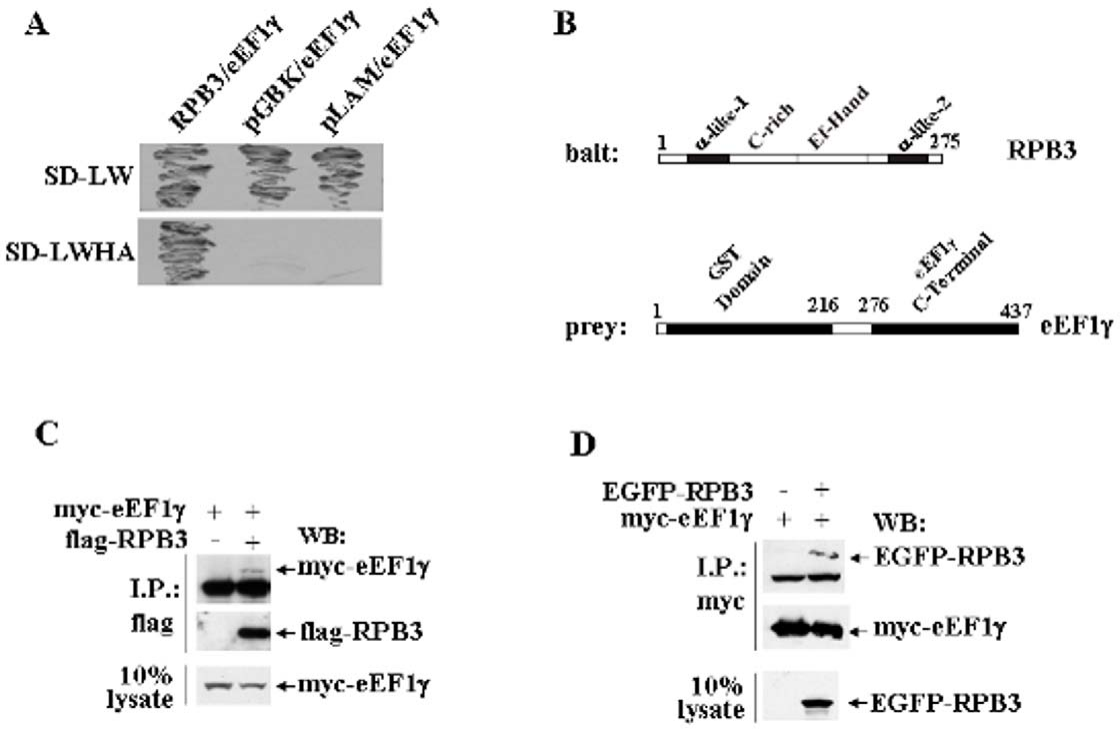
RPB3 interacts with eEF1γ. **A**: Yeast two hybrid assay: AH109 yeast cells were co-transformed with the indicated constructs and plated onto SD media lacking leucine and tryptophan (-LW) to verify the expression of both bait (W^+^) and prey (L^+^) plasmids, or onto media lacking leucine, tryptophan, histidine and adenine (-LWHA) to examine the interaction between bait and prey proteins. **B**: Schematic representation of full-length proteins of both bait (RPB3) and prey (eEF1γ). **C**: Whole cell extracts of HeLa cells transfected with either myc-eEF1γ or flag-RPB3 and myc-eEF1γ were immunoprecipitated with the anti-flag monoclonal antibody and the co-immunoprecipitation was analysed by western blot using the anti-myc monoclonal antibody. The myc-eEF1γ signal is above the heavy chain Ig band. **D**: Whole cell extracts of HeLa cells transfected with either myc-eEF1γ or with myc-eEF1γ and EGFP-RPB3 were immunoprecipitated with the anti-myc monoclonal antibody and the co-immunoprecipitation was analysed by western blot using the anti-EGFP monoclonal antibody. The EGFP-RPB3 signal is above the heavy chain Ig band.

**Figure 2:**
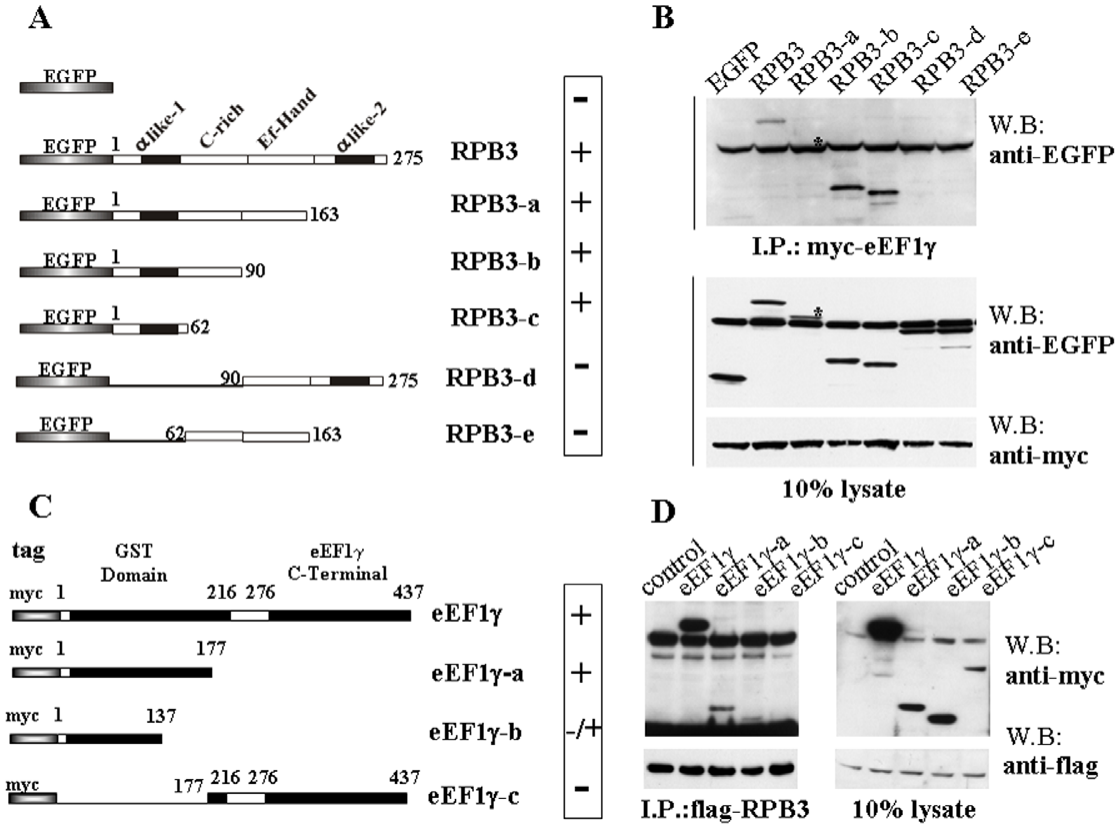
RPB3 and eEF1γ protein regions required for mutual interaction. **A**: Schematic representations of the EGFP-tagged full-length RPB3 and its derived deletion mutants. The construct designation and binding activity (to eEF1γ) are indicated on the right side. **B**: Cell lysates from HeLa cells co-transfected with each indicated construct (EGFP, EGFP-RPB3 and EGFP-RPB3 nested deletion-mutants) and myc-eEF1γ were immunoprecipitated with the anti-myc antibody. Immunoprecipitated samples were analysed by western blot using the anti-EGFP monoclonal antibody to identify RPB3 domains involved in myc-eEF1γ interaction (*top*). The total cell lysates were immunoblotted to verify the correct expression of the transfected molecules (bottom). The asterisks mark the signal corresponding to the RPB3-a mutant (lane 3), partially covered by the heavy chain Ig band (top) and a non-specific band (bottom). **C**: Schematic representations of myc-tagged full-length eEF1γ and its derived deletion mutants. Constructs designation and binding activity (to RPB3) are indicated on the right side. **D**: Cell lysates from HeLa cells co-transfected with flag-tagged RPB3 and myc-eEF1γ deletion constructs were immunoprecipitated with the anti-flag monoclonal antibody. Co-immunoprecipitation was analysed by western blot using the anti-myc monoclonal antibody (left). The total cell lysates were immunoblotted to verify the correct expression of all the transfected molecules (right).

The RPB3 subunit was used as bait (fig. 1B) in a series of yeast two-hybrid experiments to isolate RPB3-interacting proteins from a human skeletal muscle cDNA library. Of an estimated 2.5×10^6^ transformants, 30 clones proliferated on media lacking histidine and adenine as well as stained positive for β-galactosidase. Several clones were isolated for further characterisation [6], [9]–[10] and the clone encoding for the ubiquitous eukaryotic translation elongation factor 1 gamma subunit (eEF1γ) (fig. 1B) was selected for further study. The specificity of the RPB3/eEF1γ interaction was confirmed in a two-hybrid assay co-transforming eEF1γ with either RPB3, or an empty vector (pGBKT7) and a Lamin control vector (pLAM). This assay demonstrated that RPB3 interacts only with eEF1γ (fig. 1A). The eEF1γ cDNA fragment isolated in this yeast two-hybrid screen spanned almost the entire open reading frame, missing only seven amino acid residues in the extreme amino terminal portion. A full length open reading frame was isolated and cloned in the myc-tagged expression vector pCS2-MT “myc- eEF1γ” (fig. 2C and M&M section).

Co-immunoprecipitation experiments were performed to provide evidence that RPB3 also associates with eEF1γ in mammalian cells. Expression vectors for flag-tagged RPB3 (flag-RPB3) and myc- eEF1γ were co-transfected into HeLa cells. Immunoprecipitation using a flag-monoclonal antibody followed by western blot analysis of the precipitates with a myc-monoclonal antibody clearly demonstrates co-immunoprecipitation of myc-eEF1γ with flag-RPB3 (fig. 1C). These co-immunoprecipitation data were further confirmed performing the reciprocal experiment. EGFP-tagged RPB3 (EGFP-RPB3) efficiently co-immunoprecipitated with myc-eEF1γ (fig. 1D and fig. 2B).

### RPB3 and eEF1γ protein regions required for mutual interaction

Co-immunoprecipitation experiments were performed to determine the RPB3 protein region responsible for binding eEF1γ. HeLa cells were co-transfected with myc-eEF1γ and a series of RPB3 deletion mutants fused to the EGFP protein (fig. 2A) [10]. Immunoprecipitation was performed with an anti-myc monoclonal antibody and analysed by western blot using an anti-EGFP monoclonal antibody. A region 62 amino acids long at the amino terminal portion of the RPB3 protein, which corresponds to the α-like-1 domain, is required for myc-eEF1γ interaction (fig. 2B). Then, to better characterise this interaction, we decided to identify the eEF1γ protein region responsible for RPB3 binding. Thus, co-immunoprecipitation experiments in HeLa cells co-transfected with flag-RPB3 and myc-eEF1γ and a series of its deletion mutants were performed (fig. 2C–D). Immunoprecipitation using an anti-flag monoclonal antibody followed by western blot analysis with an anti-myc monoclonal antibody demonstrated that binding was greatly affected when a region 40 amino acids long between residue 137 to residue 177 was deleted (fig. 2C).

### eEF1γ interacts with pol II and binds Vimentin gene promoter region

**Figure 3:**
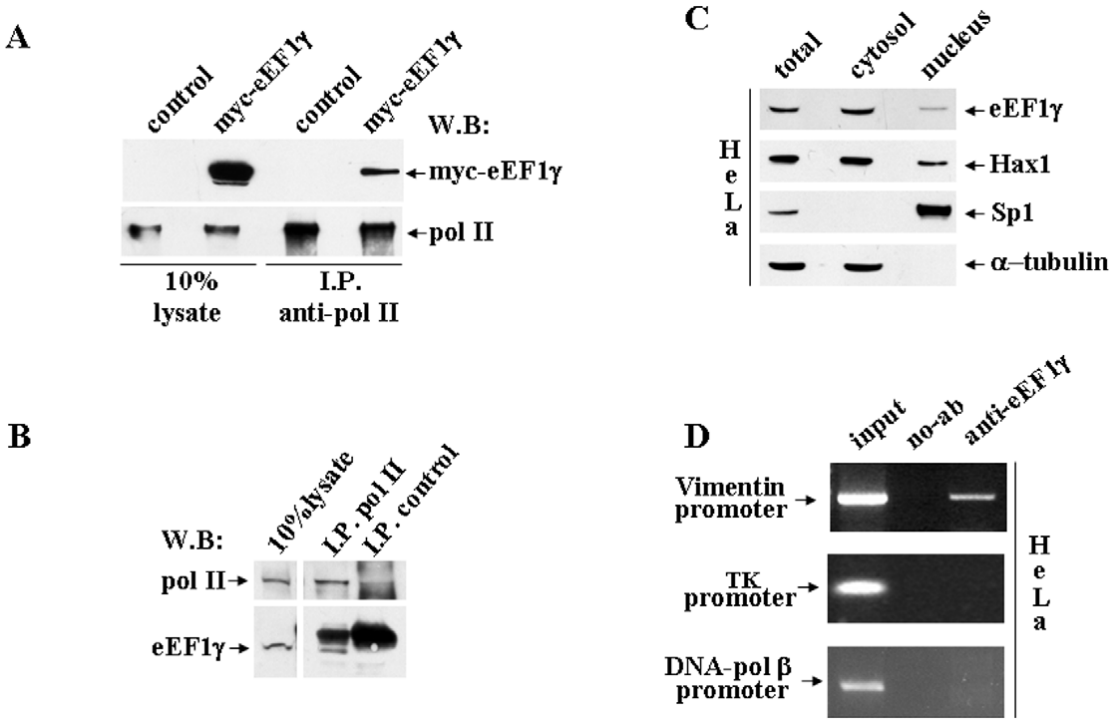
eEF1γ interacts with pol II and binds Vimentin gene promoter region. **A**: Total lysates from HeLa cells transfected with either an empty control vector or myc-eEF1γ were immunoprecipitated with the anti-pol II polyclonal antibody and analysed by western blot using the anti-myc monoclonal antibody. **B**: Whole extracts from HeLa cells immunoprecipitated with the anti-pol II polyclonal antibody. Co-immunoprecipitation was analysed by western blot using the anti-eEF1γ polyclonal antibody. The eEF1γ signal is specifically detected in the pol II I.P. sample (below the heavy chain Ig band). The I.P. control was performed with normal rabbit serum. Total cell lysates and immunopreciptated samples were immunoblotted with the anti-pol II polyclonal antibody. **C**: Western blot analysis of the cytoplasmic and nuclear fractions derived from HeLa cells. The blot was incubated with the anti-eEF1γ polyclonal antibody to determine the subcellular localization of eEF1γ. To verify fractionation quality, the same extracts were incubated with the anti-Hax1, anti-Sp1 and anti-alpha-tubulin antibodies. **D**: eEF1γ stays on Vimentin promoter at the endogenous chromosomal site. Chromatin immuno-precipitation was performed in HeLa cells using the anti-eEF1γ rabbit polyclonal antibody/protein G-agarose beads or only protein G-agarose beads as a control (no-Ab). Immuno-precipitates from each sample were analysed by PCR performed using primers specific for the human Vimentin promoter. The DNA-pol β and thymidine kinase human promoters were also amplified. A sample representing linear amplification of the total input chromatin (input) was included in the PCR as a control.

RPB3, one of the essential subunits of pol II, has most of its surface buried in interactions with other pol II subunits [27]. Yeast two-hybrid and co-immnuprecipitation assay demonstrated that the RPB3 protein alone is able to contact eEF1γ. To verify the capability of eEF1γ to contact the pol II enzyme, the total lysate from HeLa cells over-expressing myc-eEF1γ was immunoprecipitated with a polyclonal antibody against RPB1, the largest subunit of pol II (fig. 3A). Western blot analysis of the immunoprecipitates using an anti-myc monoclonal antibody clearly demonstrated the presence of myc-eEF1γ in the pol II complex. The endogenous eEF1γ protein was also able to co-immunoprecipitate with pol II. The total lysate from HeLa cells was immunoprecipitated with an anti-RPB1 polyclonal antibody followed by western blot analysis using the anti-eEF1γ polyclonal antibody, indicating the presence of eEF1γ in the pol II immunoprecipitated complex (fig. 3B). To verify the presence and possibly the relative concentration of eEF1γ in the nuclear compartment of HeLa cells, western blot analysis was performed using cytoplasmic and nuclear fractions. eEF1γ was present in both compartments in a manner resembling the pattern showed by Hax1, a principally mitochondrial, RNA-binding protein that binds Vimentin mRNA [22]. The quality of the HeLa cell fractionation was tested using anti-Sp1 and anti-α-tubulin antibodies. In summary, we showed that eEF1γ binds RPB3 and that it co-immunoprecipitates with pol II. In addition, eEF1γ was recently shown to bind 3′UTR of Vimentin mRNA [22], this region is required to perinuclear Vimentin mRNA localization [28]. The affinity of eEF1γ for Vimentin mRNA and its ability to be included in pol II complexes suggested the possibility that eEF1γ also stays on Vimentin gene promoter region. Chromatin immunoprecipitation experiments (ChIP) were performed to verify this possibility. In HeLa cells, eEF1γ is able to stay efficiently and specifically on the chromosomal locus located approximately 200 bp upstream of the Vimentin gene mRNA start site (fig. 3D). The specificity of DNA amplification was checked using the Thymidine Kinase (TK) and DNA-pol β human promoters. The results were identical when the ChIP experiments were performed on human neuroblastoma SY5Y cells (fig. S1A).

### eEF1γ depletion influences Vimentin protein localization

**Figure 4:**
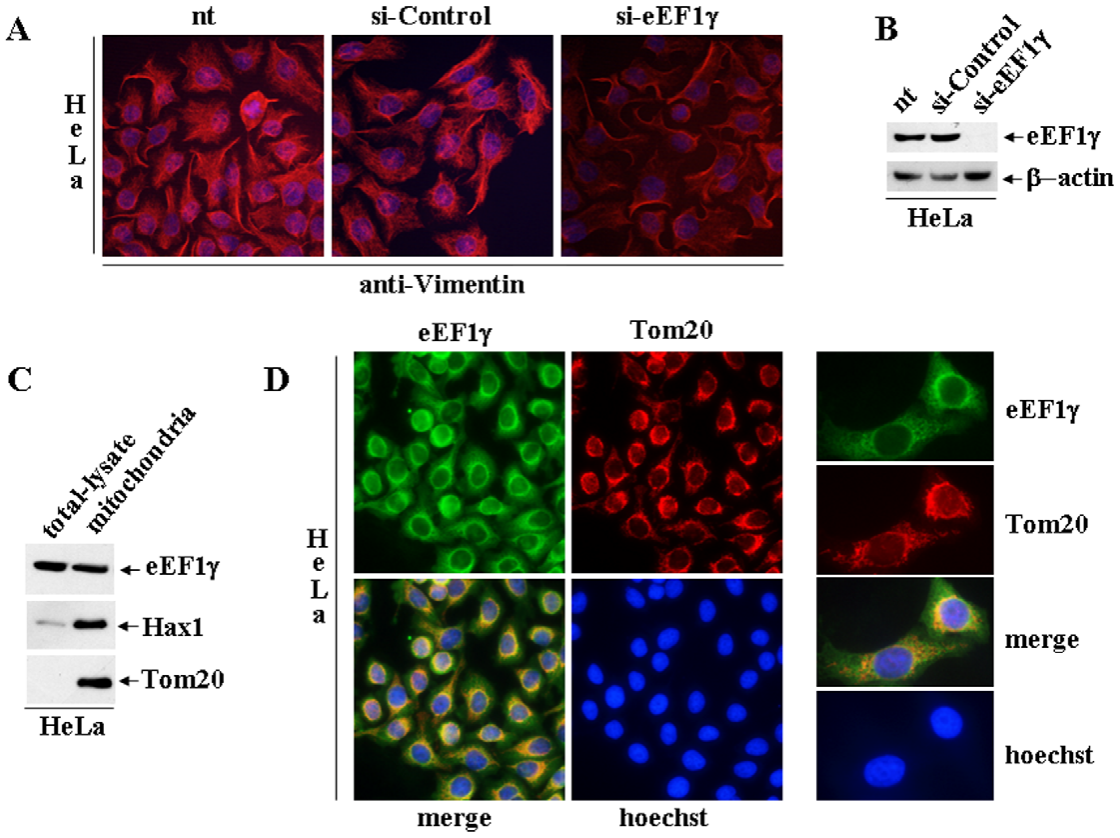
eEF1γ partially co-localizes with mitochondria and its depletion influences Vimentin protein localisation. **A**: Indirect immunofluorescence of Vimentin, obtained with the monoclonal antibody anti-Vimentin, in HeLa cells: untreated, treated with siRNA-Control and eEF1γ-depleted by specific siRNA. **B**: Western blot analysis of HeLa cells (from panel A): untreated and treated with either siRNA-Control or with eEF1γ specific siRNA. **C**: Western blot analysis of HeLa cell total lysate and the enriched mitochondrial fraction. The quality of mitochondrial enriched fraction was monitored using the anti-Hax1 monoclonal antibody and the anti-Tom20 rabbit polyclonal antibody. **D**: Dual-label indirect immunofluorescence performed in HeLa cells with the anti-Tom20 rabbit polyclonal antibody and the anti-eEF1γ monoclonal antibody to visualise the immunolocalisation of endogenous eEF1γ (green) and Tom20 (red). Extensive co-localization (yellow) between eEF1γ and Tom20 is visualised by the merged-colour image. On the right side, the same immunofluorescences are presented at a higher magnification (100×).

We then wanted to determine if the ability of eEF1γ to bind to and to compartmentalise Vimentin mRNA could influence the localisation of the Vimentin protein. HeLa cells that were untreated, treated with a scrambled siRNA-Control and treated with a siRNA specific for eEF1γ were analysed in immunofluorescence experiments using an anti-Vimentin antibody (fig. 4A). In HeLa cells depleted of eEF1γ, the Vimentin protein is mis-localised, appearing to accumulate along the cell membrane and the shapes of the HeLa cells are less organised and thick than in cells expressing eEF1γ (fig. 4A). The efficiency of eEF1γ siRNA depletion was monitored by western blot analysis (fig. 4B).

### eEF1γ partially co-localizes with mitochondria

Vimentin, an intermediate filament, is involved in structural support, signal transduction and organelle positioning. Recently, Vimentin has been shown to participate in determining mitochondrial morphology and organisation, with its knockdown resulting in mitochondrial fragmentation, swelling and disorganisation [26]. To verify if eEF1γ could influence Vimentin mitochondrial function/s, we enquired the eEF1γ mitochondrial localisation. Western blot analysis indicates the presence of eEF1γ in mitochondria-enriched HeLa cell extracts (fig. 4C). The anti-Hax1 monoclonal antibody was used to verify the quality of the cellular fractionation. Hax1 predominantly localises to mitochondria and has been shown to be able to bind Vimentin mRNA [22]. To further verify the mitochondrial localization, a rabbit polyclonal antibody against Tom20, a well-established mitochondrial marker [29], was used in a dual-label immunofluorescence assay in HeLa cells along with the anti-eEF1γ antibody. Extensive co-localisation between endogenous eEF1γ and Tom20 is revealed by the merged-colour image (fig. 4D). In addition, in figure S1B we show, in HeLa cells, the eEF1γ mitochondrial localization by merge of mitochondrion-selective dye MitoTracker (red) and indirect immunofluorescence of endogenous eEF1γ (green).

### eEF1γ siRNA depletion induces mitochondrial fragmentation and increases cellular levels of superoxide

**Figure 5:**
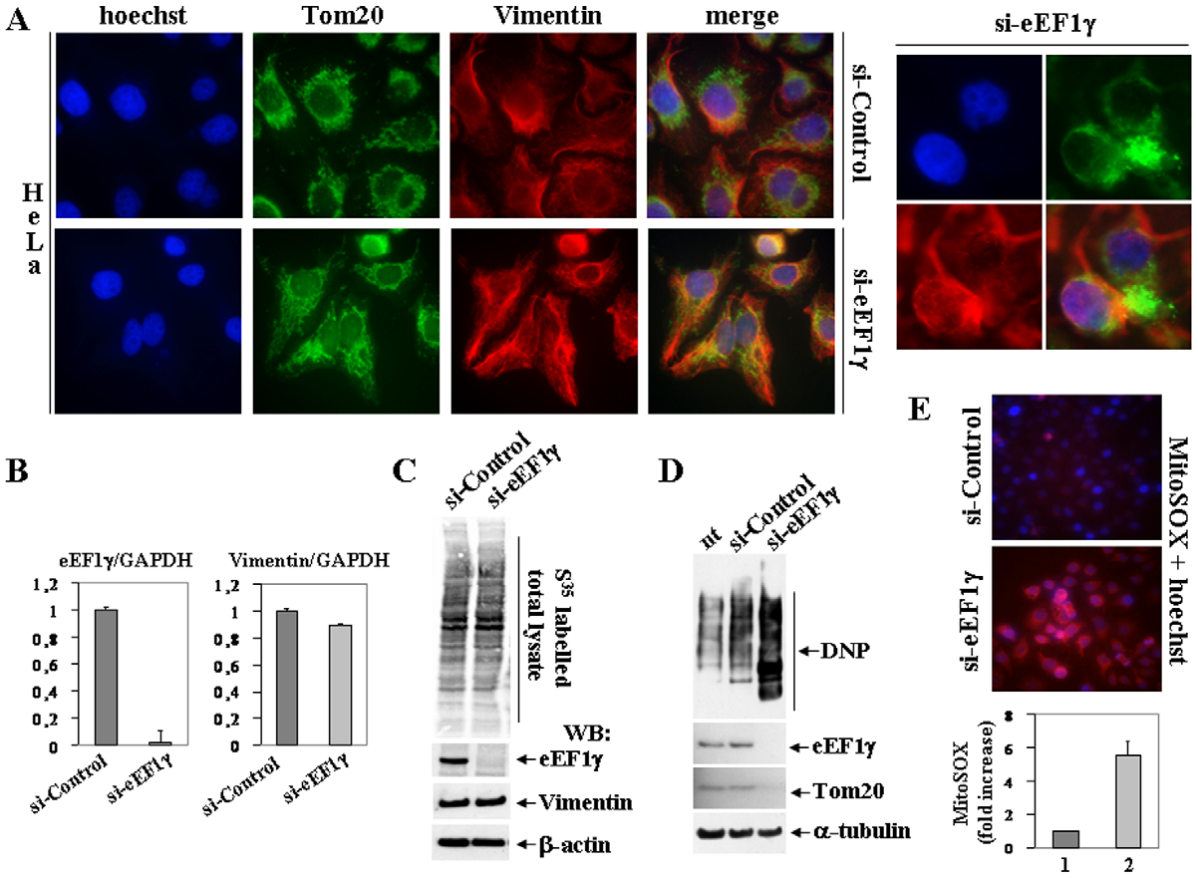
Analysis of eEF1γ depletion. **A**: Indirect immunofluorescence obtained with the anti-Tom20 rabbit polyclonal and the anti-Vimentin monoclonal antibody in HeLa cells treated with siRNA-Control or with siRNA-eEF1γ. On the right side a higher magnification image of eEF1γ-depleted HeLa cells focusing on the formation of mitochondrial dysfunctional clusters. **B**: Real time RT-PCR analysis of the eEF1γ (left) and Vimentin (right) mRNAs in HeLa cells (siRNA-Control and siRNA-eEF1γ). The gene expression ratio between eEF1γ and GAPDH and between Vimentin and GAPDH are shown as means ± SD from three independent experiments performed in triplicate. **C**: The global protein synthesis of HeLa cells, transfected with either siRNA-Control or siRNA-eEF1γ and supplemented with S^35^ labelled methionin and cystein, was visualized by autoradiography of the total protein lysates blotted to nitrocellulose membrane. Then the same membrane was incubated with the anti-eEF1γ rabbit polyclonal and the anti-Vimentin mouse monoclonal antibodies. Anti-β-actin monoclonal antibody was used to normalise the amount of protein loaded on the gel. **D**: Induced carbonylation pattern (oxidation level) of HeLa cells: untreated, treated with either siRNA-Control or siRNA-eEF1γ. The total protein carbonylation pattern was visualised by western blot with the anti-DNP antibody. Depletion of eEF1γ by siRNA was monitored using the anti-eEF1γ rabbit polyclonal antibody. The levels of Tom20 protein were monitored using the anti-Tom20 polyclonal antibody. Anti-alpha-tubulin monoclonal antibody was used to normalise the amount of protein loaded on the gel. **E**: Representative florescence images of HeLa cells treated with either siRNA-Control or siRNA-eEF1γ (top). The cellular level of superoxide was visualized by the MitoSOX (red) mitochondrial Superoxide Indicator staining. Nuclei were stained with Hoechst (blue). Histogram reporting fold of increase of MitoSOX fluorescence of siRNA-eEF1γ treated cells (lane 2) *versus* siRNA-Control treated cells (lane 1), shown as means ± SD from three independent experiments (bottom).

Using indirect immunofluorescence with both the anti-Tom20 polyclonal antibody and anti-Vimentin monoclonal antibody, HeLa cells treated with eEF1γ-specific siRNA show clear mitochondrial fragmentation, swelling and disorganisation (fig. 5A). The same effect was observed in SY5Y neuroblastoma cells (fig. S1C).

In order to investigate possible effects of eEF1γ depletion on both transcription and translation, we performed Real time RT-PCR on Vimentin transcript and methionine/cystein S^35^ total protein incorporation experiments. Histograms presented in figure 5B shows only a slight decrease of Vimentin mRNA levels upon eEF1γ knockdown in HeLa cells. Equivalent results were obtained upon eEF1γ siRNA in SY5Y cell line (data not shown). Then we investigated the effect of eEF1γ depletion on global translation. To this end we performed methionine/cystein S^35^ incorporation experiments followed by a western blot in both HeLa and SY5Y cells (fig. 5C and fig. S1D). In our experimental conditions eEF1γ-specific siRNA depletion does not affect global translation levels and in particular it does not significantly alter Vimentin translational level.

To determine if eEF1γ knockdown also induces oxidative stress, the oxidation levels of the cells were evaluated through the amount of carbonylated proteins. The total protein carbonylation pattern was visualised by western blot with an anti-DNP antibody in HeLa cells that were untreated, treated with the scrambled siRNA-control or treated with the siRNA specific for eEF1γ (fig. 5D). HeLa cells depleted of eEF1γ show a consistent increase in the levels of carbonylated proteins and a concomitant decrease in the levels of the Tom20 protein. The same effect on total protein carbonylation pattern was observed in SY5Y neuroblastoma cells (fig. S1E). In addition, a significant increase in the mitochondrial superoxide was detected in eEF1γ-depleted HeLa cells, using MitoSOX fluorescent probe (fig. 5E).

## Materials and Methods

### Yeast two-hybrid selection

For two-hybrid screening, the complete open reading frame (ORF) of human RPB3 was cloned into the BamHI restriction site of the vector pGBKT7 (Clontech, Palo Alto, CA) in frame with the *GAL4* binding domain (BD) and used to screen a human skeletal muscle cDNA library (Clontech) as previously described [9]. Yeast strain AH109 bearing UASg-His3, UASg-ADE2 and UASg-LacZ as reporter genes, was co-transformed as previously described [10]. The recovered library-derived plasmids were analysed as candidate binding partners.

### Constructs

Full-length human Flag-RPB3 (vector-pCMV-Tag2A) (Stratagene), EGFP-RPB3 (vector-pEGFP-N) (Clontech), and RPB3 deletion mutants were generated by the use of PCR amplification and/or sub-cloning [10]. The myc-tagged pCS2-eEF1γ (Myc-eEF1γ) construct and its derived deletion mutants Myc-eEF1γ-a and Myc-eEF1γ–b and Myc-eEF1γ-c were generated by PCR amplification.

### Co-immunoprecipitation and immunoblotting

Whole-cell extracts were prepared in lysis buffer (50 mM Tris-HCl pH 7.5, 250 mM NaCl, 5 mM EDTA, 0,1% Triton X-100) supplemented with a proteinase inhibitor cocktail (CompleteTM, Roche). Immunoprecipitation assays were performed following standard procedure, using the following antibodies: agarose-covalently attached anti-flag monoclonal M2 (Sigma), anti-myc monoclonal antibody (9E10 clone, hybridoma-conditioned medium) and anti-pol II rabbit polyclonal antibody (Santa Cruz). Immuno-precipitated proteins were eluted from beads by boiling in LDS Sample Buffer for 10 minutes, followed by electrophoresis on NuPAGE ® Bis-Tris Gel System (Invitrogen). Western blots were prepared by standard procedures, probed using appropriate dual immuno-staining and visualised by chemiluminescence (ECL plus; GE Healthcare) according to the manufacturer's instructions. For the biochemical analysis, the following antibodies were used: anti-myc monoclonal antibody (9E10 clone, hybridoma-conditioned medium), anti-flag rabbit polyclonal antibody (Sigma), anti-pol II rabbit polyclonal antibody (Santa Cruz), anti-eEF1γ rabbit polyclonal (Bethyl Laboratories Inc.) and anti-GFP monoclonal antibody (Clontech).

*Immunofluorescence-* Immunofluorescence was performed to detect the Vimentin and Tom20 proteins in HeLa and SY5Y cells. The cells were transfected with siRNA Dharmacon and 72 hours later were fixed/permeabilised in 3∶7 methanol-acetone for 20 min at −20°C or fixed with 4% paraformaldheyde for 10 min at R.T. and then permeabilised with 0.2% Nonidet P-40 in phosphate-buffered saline (PBS) for 10 minutes. To detect the expression of endogenous Vimentin and/or Tom20, the cells were incubated for 1 hour at room temperature with a 1∶50 dilution (in PBS containing 2% BSA) of mouse monoclonal anti-Vimentin (Dako) or with a 1∶200 dilution of rabbit polyclonal anti-Tom20 antibody (Santa Cruz). Immunoreactivity was visualised using an Alexa-Fluor594-conjugated anti-mouse IgG (Molecular Probes, Invitrogen) secondary antibody or an Alexa-Fluor488-conjugated anti-rabbit IgG (Molecular Probes, Invitrogen) secondary antibody. To detect co-localisation of eEF1γ and Tom20 proteins, HeLa cells were fixed in 4% paraformaldehyde for 10 min at R.T., permeabilised with 0.2% Nonidet P-40 in PBS for 10 minutes and incubated overnight with the anti-eEF1γ mouse monoclonal antibody (1∶50 dilution) (Abnova Corporation), plus one additional hour together with anti-Tom20 rabbit polyclonal antibody.

To label mitochondria, HeLa cells were incubated with 250 nM of MitoTracker® Red CMXRos M7512 (Invitrogen) according to the manufacture's instructions. Once the mitochondria were labelled, the cells were fixed and incubated overnight with the anti-eEF1γ mouse monoclonal antibody, as above.

### Mitochondrial superoxide detection

Mitochondrial superoxide generation was detected using MitoSOX™ Red (Molecular Probes, Invitrogen), a specific mitochondrial superoxide indicator of reactive oxygen species (ROS), according to manufacturer protocol. Briefly, HeLa cells were incubated in 5 µM MitoSOX in PBS for 10 min followed by staining with Hoecst (4 µg/ml). After incubation, cells were washed in PBS and examined under inverted microscope (LEICA FW4000). For semiquantification of MitoSOX fluorescence, five non adjacent images were taken for each group under identical exposure condition and cell numbers (Hoecst positive nuclei) in each image were counted. Data from a total of three independent experiments were analysed. MitoSOX fluorescence was quantified using ImageJ analysis software and normalized for cell counts.

### Cell culture and transfections

Human HeLa cells (ATCC, CCL-2) were grown in Dulbecco's modified Eagle's medium (DMEM) supplemented with 10% foetal bovine serum (FBS; Gibco). Human SY5Y neuroblastoma cells (ATCC, CRL-2266) were grown in DMEM supplemented with 15% foetal bovine serum. All cell cultures were maintained at 37°C in a humidified atmosphere of 5% CO_2_. Transient transfections were performed using Lipofectamine or Lipofectamine 2000 reagents (Invitrogen), according to the manufacturer's instructions. The total amounts of transfected DNA were equalised using empty vectors.

The siRNA-mediated interference experiments for eEF1γ expression were performed by transfecting SMART pool-specific or non-specific control pool double-stranded RNA oligonucleotides (Dharmacon) using Lipofectamine 2000.

### Sub-cellular fractionation

HeLa cells (∼6×10^6^) were rinsed three times with ice-cold PBS, harvested, and centrifuged at 1200 g for 2 min at 4°C. The cell pellet was lysed in 200 µl of buffer A (50 mM Tris-HCl pH 7.5, 5 mM EDTA, 10 mM NaCl, 0.05% NP40, 0.5 mM DTT, protease inhibitor cocktail) and centrifuged for 30 seconds at 15000 g at 4°C. The supernatant was saved as the cytoplasmic fraction. The pellet was rinsed two times with buffer A. The nuclear pellet was lysed in 65 µl of buffer B (20 mM Hepes pH 7.9, 1.5 mM MgCl_2_, 0.2 mM EDTA, 420 mM NaCl, 25% glycerol, protease inhibitor cocktail), incubated on ice for 30 min and centrifuged for 10 min at 15.000 g at 4°C. The supernatant was saved as the nuclear fraction. The mitochondrial fraction was purified from HeLa cells (∼2×10^7^) using the Qproteome Mitochondria Isolation Kit (Qiagen) according to the manufacturer's instructions. All fractions were reconstituted to a final concentration of 1X LDS Sample Buffer and analysed by the NuPAGE ® Bis-Tris Gel System (Invitrogen). The purity of the cytosolic, nucleic and mitochondrial fractions, was determined by probing with anti-α-tubulin monoclonal antibody (Merck4Biosciences), anti-Sp1 monoclonal antibody (Santa Cruz) and anti-Hax1 monoclonal (BD Biosciences)/anti-Tom20 polyclonal (Santa Cruz) antibodies, respectively.

### Chromatin Immunoprecipitation (ChIP) assay

Chromatin Immunoprecipitation assay was performed as previously described [30]. Briefly, HeLa or SY5Y cells (∼2×10^7^) were cross-linked with 1% formaldehyde for 10 min at 37°C and lysed. The cell lysate was sonicated on ice, resulting in DNA fragments approximately 500 bp in length. Equal amounts of chromatin from each sample were immunoprecipitated over night with the anti-eEF1γ rabbit polyclonal antibody (Bethyl Laboratories, Inc.). Immunoprecipitation with no specific immunoglobulins (Santa Cruz) was performed as a negative control. DNA representing 0.005% of the sonicated chromatin solution (input) and 10% of the immunoprecipitated sonicated chromatin solution were amplified using the human Vimentin specific primers, hVIM forward (5′-CCGCAGCCCCGAGACCGCCGCGCA-3′) and hVim reverse (5′-GTCCCGTTACTTCAGCGCTGGGCT-3′), the human thymidine kinase specific primers, hTK forward (5′-GCCCCTTTAAACTTGGTGGGCGG-3′) and hTK reverse (5′-TTGCGCCTCCGGGAAGTTCACG-3′), the DNA polymerase β specific primers, hPolβ forward (5′-TCAGAATCAAGATCGCACTCCCGT-3′) and hPol β reverse (5′-GCGCTTGTTGTGACGTCACGCGTCC-3′). PCR conditions were: 30 cycles at 95°C for 45 s, 60-67°C for 30 s, 72°C for 30 s and a final extension at 72°C for 5 min.

### RNA extraction, retrotranscription and real-time PCR

Total RNA was extracted from HeLa cells transfected with siRNA Dharmacon using TRIzol reagent according to the manufacturer's instructions (Invitrogen). 2 µg of RNA was reverse transcribed using oligo (dT) _12–18_ primers and Superscript II (Invitrogen) in a final volume of 20 µl, at 42°C for 50 min. A real-time PCR assay was performed in a 96-well format using the ABI Prism 7000 Sequence Detection System (Applied Biosystems, Foster City, CA). To obtain the vimentin or eukaryotic translation elongation factor 1 gamma gene expression rate the amount of target genes were normalized to that of the housekeeping gene Glyceraldehyde 3-phosphate dehydrogenase (GAPDH). Primers and probes for VIM or eEF1γ (target genes) and for GAPDH (housekeeping gene) were purchased as TaqMan Gene Expression Assays (Applied Biosystems). The PCR mixtures containing the cDNA template, the TaqMan Universal PCR master mix (AB) and the primers/probe were analysed in triplicate using standard Taqman conditions. The results were analyzed using Applied Biosystems analysis software. The data are expressed as the ratio between VIM or eEF1γ and GAPDH mRNAs expression.

### S35-Labeling Experiments

HeLa or SY5Y cells were transfected with siRNA Dharmacon and 72 hours later were starved for 1 hour and 30 min in DMEM lacking Met/Cys. [S^35^]Met/[S^35^]Cys (PerkinElmer Life Sciences) were added to the medium (44 µCi/ml; 1 Ci/37 GBq), and the cells were incubated for further 3 hours. The cell lysates were then subjected to SDS-PAGE, transferred to a nitrocellulose membrane and detected by autoradiography. The membrane was then incubated with the following antibodies: the anti-Tom20 rabbit polyclonal, the anti-Vimentin mouse monoclonal (Sigma) and the anti-β-actin monoclonal.

### Protein Carbonyl Assay

Protein carbonyl levels, an index of protein oxidation, were determined by using the Oxyblot Protein Oxidation Detection kit (Chemicon) according to the manufacturer's instructions. Briefly, 15–20 µg of protein were diluted to a final concentration of 6% SDS and incubated with 2,4-dinitrophenylhydrazine (DNPH) for 15 min. Then, samples were neutralized, electrophoresed on NuPage gels (Invitrogen), transferred and blocked. The membrane was first incubated with the anti-DNP rabbit polyclonal antibody (1∶150), then stripped and subsequently reprobed for anti-α-tubulin and anti-Tom20 antibodies.

## Discussion

The novelty presented in this manuscript is that eEF1γ, a subunit of the elongation factor-1 complex, interacts with pol II and binds Vimentin gene promoter, where eEF1γ may start to play its function/s. We initially isolated eEF1γ in a yeast two-hybrid screening using RPB3, the α-like RNA polymerase II core subunit, as bait. The eEF1γ characterization as RPB3 protein partner was intriguing and we started the analysis considering that both bait “RPB3” and pray “eEF1γ” appear extremely conserved during evolution.

The bacterial alpha subunit homodimer directly interacts with sigma factors enabling the specific binding of RNA polymerase to gene promoters. The eukaryotic alpha subunit homodimer is highly conserved and can be recognised in the two α-like RNA polymerase II core subunits RPB3 and RPB11. In their precious paper, Fan et al. (2010), described Drosophila eEF1γ essential for organismal and cellular viability [18], whereas the yeast eEF1γ orthologue has been defined as a non essential gene [31]. The Biological General Repository for Interaction Datasets (Biogrid) from yeast to human reveals a large number of potentially interacting proteins for eEF1γ, mostly of which are involved in RNA metabolism, transcription regulation or mitochondrial metabolism. Some genes of particular interest are the following: yeast She2, a RNA-binding protein that is part of the mRNA localisation machinery that restricts the accumulation of certain proteins to the bud [32]; yeast Whi3, a RNA binding protein that sequesters CLN3 mRNA in cytoplasmic foci [33]; TAF2, a TFIID subunit involved in pol II transcription initiation; YSP1 (Yeast Suicide Protein), a mitochondrial protein with a potential role in promoting mitochondrial fragmentation during programmed cell death [34]; human Med31, a component of the Mediator complex and a co-activator involved in the regulated transcription of nearly all pol II-dependent genes [35] and human Nup85, an essential component of the nuclear pore complex involved in RNA export [36]. We showed that the RPB3 and eEF1γ proteins co-immunoprecipitate when over-expressed in HeLa cells (and in SY5Y cells; data not shown). To better understand the nature of the RPB3/eEF1γ interaction, we defined the RPB3 and eEF1γ protein regions responsible for reciprocal binding. The α-like-1 domain, a 62 amino acids region at the amino terminal of RPB3, appears to be responsible for this interaction. This region is also in charge to bind both RPB11, the second α-like pol II subunit [6], [37]–[38] and CCHCR1 which compartmentalises RPB3 in the cytosol [10]. The observation that eEF1γ requires the same region that RPB3 uses to bind RPB11 inside the pol II enzyme is intriguing. This finding opens the interesting possibility that eEF1γ could be an alternative to RPB11 in the pol II assembly. This issue will be a focus of our future work. On eEF1γ side, we defined a crucial RPB3-interacting region 40 amino acid long between residue 137 to residue 177. This eEF1γ region apparently does not share homology with other proteins described to bind RPB3.

Taking into account that RPB3 protein exerts its crucial role in the nucleus as part of the pol II core enzyme, we demonstrated that eEF1γ co-immunoprecipitates with the pol II enzyme. Fan et al. (2010) showed that eEF1γ is detectable in Drosophila pupal nuclear extracts and we confirmed the presence of human eEF1γ in HeLa cell nuclear extracts [18]. Hax1, another Vimentin mRNA binding protein, was also shown to be present in HeLa cell nuclear extracts. Hax1 is a mitochondrial protein present also in the cytosol and in the nucleus. Hax1 deficiency causes autosomal recessive severe congenital neutropenia (SCN), or Kostmann disease [39]–[40]. Hax1 is involved in signal transduction, cytoskeletal control, mRNA transport and programmed cell death and is critical to maintaining the inner mitochondrial membrane potential [39], [41]. Both Hax1 and eEF1γ have the ability to bind Vimentin mRNA and have similar cellular localisations. Based upon the presence of eEF1γ in the nuclear compartment, its ability to bind RPB3 and pol II plus its aptitude to combine with Vimentin mRNA we investigated the possibility that eEF1γ could also stay on the Vimentin gene promoter. Chromatin immunoprecipitation (ChIP) experiments were used to examine the Vimentin promoter region immediately adjacent to its transcription start site. eEF1γ appears to bind Vimentin promoter region in both HeLa and SY5Y human cell lines. Based on a parallel between eEF1γ and Hax1 proteins, we also analyzed the promoter region of the DNA-polβ gene, whose mRNA is complexed with Hax1 [42]. In our experimental conditions eEF1γ did not bind DNA polβ gene promoter, whereas we demonstrate that eEF1γ binds the promoter region of the Che-1/AATF gene [43]–[44], another pol II binding protein (data not shown). It emerges that eEF1γ controls Vimentin gene through different pathways. Al-Maghrebi et al. (2002) showed that eEF1γ and Hax1 proteins bind the 3′ untraslated region (3′UTR) of Vimentin mRNA [22], while Bermano et al. demonstrated that the 3′UTR of Vimentin mRNA is required for Vimentin RNA perinuclear localization [28]. Morris et al. (2000) elegantly demonstrated that misdirecting Vimentin mRNA it is possible to alter cell morphology and motility [25]. They demonstrated it replacing Vimentin 3′UTR, where the Hax1 and eEF1γ binding sites are present, with β-actin 3′-UTR. Here, we show that depletion of eEF1γ by siRNA induces Vimentin protein de-localisation, Vimentin appears to accumulate along cell membrane and cellular shape comes out less organized and thick. Vimentin is a member of the intermediate filament family of proteins, it is important for obtaining cellular cytoskeleton flexibility [45]. Vimentin also plays a significant role in supporting and anchoring the position of the organelles in the cytosol supporting mitochondrial morphology and organization [26]. Following this rationale, we verified the presence of eEF1γ within mitochondria in human cells. We demonstrated its presence in an enriched HeLa mitochondria extract using both Hax1 and Tom20 proteins as mitochondrial markers. Of interest Tom20 was recently shown to mediate the localisation of mRNAs to mitochondria [46]. Indirect immunofluorescence also revealed the partial mitochondrial co-localisation of eEF1γ with both Tom20 and the mitochondrion-selective dye, MitoTracker. To further examine the potential role of eEF1γ in mitochondrial metabolism, mitochondria were examined after eEF1γ depletion by siRNA. Indirect immunofluorescence of Tom20 in HeLa and SY5Y cells showed clear mitochondrial fragmentation, swelling and disorganisation. This mitochondrial crisis is accompanied by a dramatic increase in the levels of superoxide and a consistent increase in the levels of carbonylated proteins. We tested the impact of eEF1γ depletion on Vimentin gene expression detecting a slight decrease in mRNA level without any significant change in protein level. In addition, we did not find any impact of eEF1γ depletion on global translation. These data are in agreement with the finding that depletion of eEF1γ in the yeast *Saccharomices cerevisiae* results in resistance to oxidative stress without any detectable alterations in total protein synthesis [14]. Moreover a very recent work shows that Yeast strains lacking eEF1γ accumulate a greater amount of oxidized proteins which correlates with changes in heat shock chaperones and altered vacuole morphology [47]. All these data taken together consent to speculate that eEF1γ can be involved in stress response and that its depletion impairs a proper cell reaction to specific stress events. On the other hand, the role of eEF1γ in translation is suggested principally by its co-purification with the elongation factor-1 complex. Several studies concerning eEF1γ function/s have as common denominator a broader role of this protein in RNA and protein metabolisms.

Our on going work is focused on isolation and characterization of novel mRNAs bound to eEF1γ protein. Here, we suggest that eEF1γ in addition to its role in translation elongation, participates in governing specific genes in certain stress conditions by contacting both pol II and gene promoters and then shuttling/nursing mRNAs, as Vimentin mRNA, from a gene locus to their appropriate cellular compartment for translation.

## Supporting Information

Figure S1 A: eEF1γ binds the Vimentin promoter at the endogenous chromosomal site. Chromatin immuno-precipitation was performed in human neuroblastoma SY5Y cell line using the anti-eEF1γ rabbit polyclonal antibody/protein G-agarose beads or only protein G-agarose beads as a control (no-Ab). Immuno-precipitates from each sample were analysed by PCR performed using primers specific for the human Vimentin promoter. DNA-pol β and thymidine kinase human promoters were also amplified. A sample representing the linear amplification of the total input chromatin (input) was included in the PCR as a control. B: Dual-label immunofluorescence in HeLa cells to show the co-immunolocalisation of endogenous eEF1γ (green) and mitochondria using the anti-eEF1γ monoclonal antibody and the mitochondrion-selective dye MitoTracker (red). C: Indirect immunofluorescence obtained with the anti-Tom20 rabbit polyclonal antibody in SY5Y cells: treated with siRNA-Control or siRNA-eEF1γ. D: The global protein synthesis of SY5Y cells, transfected with either siRNA-Control or siRNA-eEF1γ and supplemented with S35 labelled methionin and cystein, was visualized by autoradiography of the total protein lysates blotted to nitrocellulose membrane. Then the same membrane was incubated with the anti-eEF1γ rabbit polyclonal and the anti-Vimentin monoclonal antibodies. Anti-β-actin monoclonal antibody was used to normalise the amount of protein loaded on the gel. E: The induced carbonylation pattern (oxidation level) of human neuroblastoma SY5Y cell line treated with either siRNA-Control or siRNA-eEF1γ. The total protein carbonylation pattern was visualised by western blot with the anti-DNP antibody. Depletion of eEF1γ by siRNA was monitored using the anti-eEF1γ rabbit polyclonal antibody. Vimentin protein level was monitored by anti-Vimentin monoclonal antibody. Anti-alpha-tubulin monoclonal antibody was used to normalise the amount of protein loaded on the gel.
(10.73 MB TIF)
