# Supplementary figures and images for: The eEF1γ Subunit Contacts RNA Polymerase II and Binds Vimentin Promoter Region
Source: PLoS One. 2010 Dec 31;5(12):e14481. doi: 10.1371/journal.pone.0014481 (PMC3013090; doi:10.1371/journal.pone.0014481)

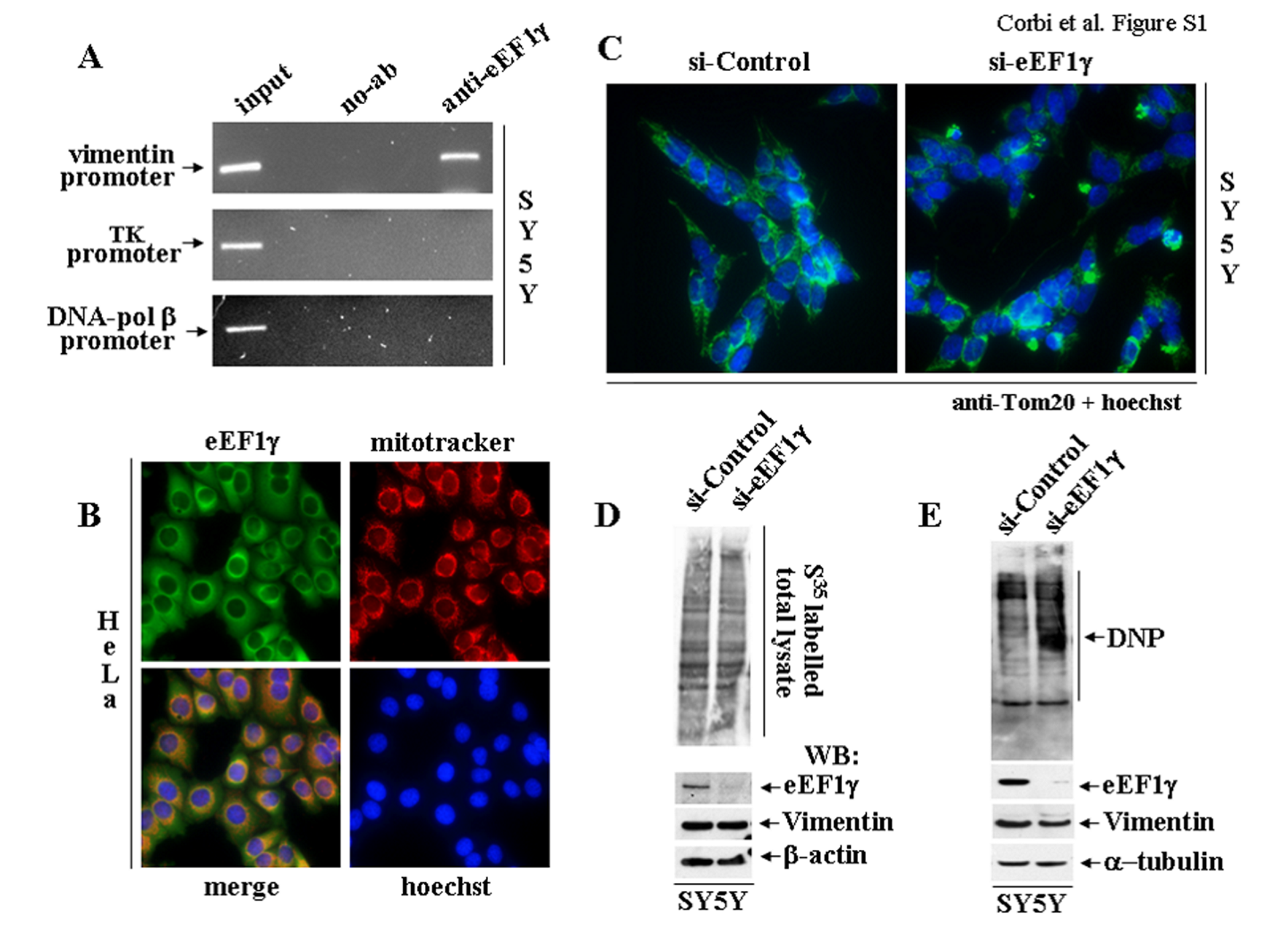

Supplement: Figure S1 — A: eEF1γ binds the Vimentin promoter at the endogenous chromosomal site. Chromatin immuno-precipitation was performed in human neuroblastoma SY5Y cell line using the anti-eEF1γ rabbit polyclonal antibody/protein G-agarose beads or only protein G-agarose beads as a control (no-Ab). Immuno-precipitates from each sample were analysed by PCR performed using primers specific for the human Vimentin promoter. DNA-pol β and thymidine kinase human promoters were also amplified. A sample representing the linear amplification of the total input chromatin (input) was included in the PCR as a control. B: Dual-label immunofluorescence in HeLa cells to show the co-immunolocalisation of endogenous eEF1γ (green) and mitochondria using the anti-eEF1γ monoclonal antibody and the mitochondrion-selective dye MitoTracker (red). C: Indirect immunofluorescence obtained with the anti-Tom20 rabbit polyclonal antibody in SY5Y cells: treated with siRNA-Control or siRNA-eEF1γ. D: The global protein synthesis of SY5Y cells, transfected with either siRNA-Control or siRNA-eEF1γ and supplemented with S35 labelled methionin and cystein, was visualized by autoradiography of the total protein lysates blotted to nitrocellulose membrane. Then the same membrane was incubated with the anti-eEF1γ rabbit polyclonal and the anti-Vimentin monoclonal antibodies. Anti-β-actin monoclonal antibody was used to normalise the amount of protein loaded on the gel. E: The induced carbonylation pattern (oxidation level) of human neuroblastoma SY5Y cell line treated with either siRNA-Control or siRNA-eEF1γ. The total protein carbonylation pattern was visualised by western blot with the anti-DNP antibody. Depletion of eEF1γ by siRNA was monitored using the anti-eEF1γ rabbit polyclonal antibody. Vimentin protein level was monitored by anti-Vimentin monoclonal antibody. Anti-alpha-tubulin monoclonal antibody was used to normalise the amount of protein loaded on the gel. (10.73 MB TIF) [file pone.0014481.s001.tif]
